# Non-Invasive Estimation of Right Atrial Pressure Using a Semi-Automated Echocardiographic Tool for Inferior Vena Cava Edge-Tracking

**DOI:** 10.3390/jcm11123257

**Published:** 2022-06-07

**Authors:** Luca Mesin, Piero Policastro, Stefano Albani, Christina Petersen, Paolo Sciarrone, Claudia Taddei, Alberto Giannoni

**Affiliations:** 1 Mathematical Biology and Physiology, Department Electronics and Telecommunications, Politecnico di Torino, 10129 Torino, Italy; piero.policastro@polito.it; 2 SC Cardiologia Ospedale Regionale U. Parini, 11100 Aosta, Italy; albani.aosta@gmail.com; 3 Fondazione Toscana G. Monasterio, 56124 Pisa, Italy; petersen@ftgm.it (C.P.); paolo.sciarrone@gmail.com (P.S.); c.taddei85@gmail.com (C.T.); or a.giannoni@santannapisa.it (A.G.); 4 Scuola Superiore Sant’Anna, 56127 Pisa, Italy

**Keywords:** inferior vena cava, ultrasound imaging, pulsatility, fluid volume assessment, right atrial pressure

## Abstract

The non-invasive estimation of right atrial pressure (*RAP*) would be a key advancement in several clinical scenarios, in which the knowledge of central venous filling pressure is vital for patients’ management. The echocardiographic estimation of RAP proposed by Guidelines, based on inferior vena cava (IVC) size and respirophasic collapsibility, is exposed to operator and patient dependent variability. We propose novel methods, based on semi-automated edge-tracking of IVC size and cardiac collapsibility (cardiac caval index—CCI), tested in a monocentric retrospective cohort of patients undergoing echocardiography and right heart catheterization (RHC) within 24 h in condition of clinical and therapeutic stability (170 patients, age 64 ± 14, male 45%, with pulmonary arterial hypertension, heart failure, valvular heart disease, dyspnea, or other pathologies). IVC size and CCI were integrated with other standard echocardiographic features, selected by backward feature selection and included in a linear model (LM) and a support vector machine (SVM), which were cross-validated. Three RAP classes (low < 5 mmHg, intermediate 5–10 mmHg and high > 10 mmHg) were generated and RHC values used as comparator. LM and SVM showed a higher accuracy than Guidelines (63%, 71%, and 61% for LM, SVM, and Guidelines, respectively), promoting the integration of IVC and echocardiographic features for an improved non-invasive estimation of RAP.

## 1. Introduction

Among the different imaging modalities available in cardiology, echocardiography is surely the most useful tool when hemodynamic assessment of the cardiovascular system is taken into account [1,2,3,4]. By exploiting basic principles of physics and different equations, ultrasounds (US) have progressively replaced cardiac catheterization when evaluating the severity of valvular heart disease [2,5]. Likewise, echocardiography has long been used (still with some imprecision) to non-invasively estimate pulmonary artery pressure [6,7,8] or cardiac output [9]. The prediction of left ventricular filling pressures has been more challenging, with heterogenous performance of different (semiquantitative or quantitative) algorithms and variable numbers of undetermined cases [10,11,12]. Recently, equations have been developed to calculate vascular pulmonary resistances [12,13] or to distinguish between precapillary or postcapillary pulmonary hypertension [14,15,16] in an overall effort to decrease the need of invasive hemodynamic assessment.

One of the highest imprecisions in the prediction of hemodynamic measures by echocardiography is observed in the assessment of right atrial pressure (RAP) [17,18,19,20]. The knowledge of RAP is clinically relevant, since it may help to stratify prognosis in both patients with heart failure [21,22] and pulmonary hypertension [8,23], and it is vital to optimize fluid management (loading and unloading) in several clinical scenarios [24]. *RAP* is currently calculated by measuring inferior vena cava (IVC) dimension and collapsibility during respiration (indicated as caval index or collapsibility index) [7]. Despite its wide use, echocardiographic estimation of *RAP* may largely differ from the invasive measure [25,26]. Sources of imprecision are [25,26]: (a) variability in the IVC sampling position; (b) displacement of IVC during inspiration with misalignment of echocardiographic original plane; (c) variability in the individual inspiratory maneuver; and (d) lack of any other left and right ventricular hemodynamic variables potentially influencing RAP in the current algorithm.

Recently, our group has developed a semi-automated method to delineate and track displacements of the IVC borders in long [27] or short axis views [28]. In preliminary studies, integrating the outputs of both methods showed promising results, providing a more reliable estimation of the volemic status of patients than using the standard IVC assessment [29]. Some attempts to estimate the RAP were also performed [30,31], but in a small cohort of patients. In the current study, we aimed at developing novel linear and non-linear algorithms for *RAP* estimation, using a larger dataset and incorporating, beyond semi-automated tools for IVC edge tracking, also other key echocardiographic variables, in a population of patients undergoing right heart catheterization (RHC).

## 2. Materials and Methods

### 2.1. Experimental Data

In the current study a series of patients in condition of clinical stability undergoing echocardiography (Philips iE33 xMATRIX echocardiography system, Andover, MA, USA) and RHC for any clinical indication within 24 h from echocardiography at Fondazione Toscana G. Monasterio (FTGM, Pisa, Italy) were retrospectively analyzed. No changes in patients’ medication of fluid therapy was allowed between echocardiography and RHC. Exclusion criteria were duration of the US video < 1 s or poor echocardiographic image quality (i.e., shadow cones on echocardiographic windows interfering with IVC visualization).

Due to the retrospective nature of the study, we did not ask for an approval of the Ethics Committee: the feasibility assessment was internal to our Institute. In fact, the patients data were acquired for clinical reasons; they did not carry out a research protocol, but they signed consent for the execution of the right heart catheterization and for the possible use of the recorded data for scientific purposes. An ID code was assigned to each patient, in line with privacy policy.

Echocardiography and RHC were performed according to International Recommendations [1,7,10,32], as described in detail in the Appendices Appendix A and Appendix B. IVC was measured along its long axis in 2D images in the subcostal view, with the patient in the supine position at 1–2 cm from the junction of the right atrium. The diameter of IVC and the percentage decrease during inspiration (brief sniff), or caval index were calculated and RAP was estimated according to guidelines [7]: IVC with diameter < 2.1 cm and that collapses > 50% with a sniff suggests normal RAP (range, 0–5 mmHg); IVC with diameter > 2.1 cm and that collapses < 50% with a sniff suggests high RAP (range, 10–20 mmHg); intermediate values (range, 5–10 mmHg) are assumed in the other cases [18,33].

Beyond standard echocardiographic assessment, a non-invasive estimation of pulmonary artery wedge pressure (PAWP) was completed, using a previously validated equation, which includes the following variables [12]: tricuspid regurgitation velocity (TRV), left ventricular ejection fraction (LVEF), right ventricle fractional area change (FAC), left atrial volume index (LAVi), E/E′, IVC, and mean pulmonary artery pressure (mPAP). Then, pulmonary vascular resistance (PVR) was calculated as (mPAP−PAWP)/CO, where CO is the cardiac output [12].

### 2.2. Processing of Ultrasound Scans with Edge-Tracking Technique

The echocardiographic video clips were exported in DICOM format and then sent via a cloud platform to P.P. to perform the processing. The borders of a section of the IVC were identified automatically, using the algorithm proposed in [27] and used in different previous applications [29,30,31,34]. The processing method is semi-automated: the user indicated different information for the processing in the first frame of the video, e.g., two reference points to track (in order to compensate IVC movements) and the portion of interest. Each frame was then smoothed with a 2D median filter and the software computed the IVC edges in the region of interest sampled on 30 points (edge points found as jumps of intensity). From the two IVC borders, the IVC median line and 5 diameters orthogonal to it were computed, so as to cover 15 mm of the central part of the portion of interest (thus avoiding extremities). Pulsatility was assessed along each of these diameters in terms of the cardiac caval index (CCI) [35], as the US videos were short (in the range 1–3 s), so that respirophasic collapsibility could not be measured. The CCIs were measured along each diameter and for each heartbeat within the considered US scan. All those values were averaged, to obtain a stable characterization of the pulsations of the IVC induced by the heartbeats. Moreover, the mean diameter was computed averaging across different diameters and frames, to obtain an estimation of IVC size.

### 2.3. Estimation of Right Atrial Pressure

RAPs of patients were split into three classes: low (≤5 mmHg), intermediate (>5, ≤10 mmHg), and high (>10 mmHg), as suggested by the Guidelines [1,7]. RAP was estimated considering two approaches: support vector machine (SVM) with a radial basis function kernel and linear model (LM), the first estimating each class (by a one versus all approach) and the latter computing continuous values (that were then also split into 3 classes to compare it to the other method). These models were built to estimate the RAP, selecting the best echography features of our dataset, in addition to information extracted from US scans of IVC. Specifically, we included the mean diameter and 1/(CCI+a), with a=0.3, as in [31], where this latter function of CCI was found to be maximally correlated to RAP. Different features have been recorded by the medical doctors. Due to the retrospective nature of this work, we established that only echocardiographic variables that were present in more than 95% of subjects were included in the algorithm. To the missing values, we assigned the medians of the distributions of the available measurements. Table 1 reports the list of features that were included in this study, together with the number of missing values.

Backward feature selection was used to identify subsets of features that allow the models to obtain maximal accuracy, but with the minimum number of essential inputs. Specifically, one feature at a time was removed iteratively from the dataset. The cross-validation accuracy was computed for each subset (k-fold cross-validation with k = 10), selecting the one for which it was maximal, thus excluding the less informative feature. Iterating the procedure, the least important feature was removed for each iteration, until a single feature (i.e., the most important) remained. In this way, the features were selected in order of importance. This allows the simplest model still providing good accuracy to be identified, solving the curse of dimensionality and avoiding overfitting. As mentioned above, this procedure was applied separately to the two models: *SVM* and *LM* (the latter making a classification imposing thresholds at 5 and 10 mmHg to split the estimated continuous values of *RAP* into 3 classes).

The best models were finally cross-validated by leave one out and compared to the estimations obtained by guidelines, using the manual measurements of the caval index and diameter.

### 2.4. Statistical Analysis

A preliminary exploration of the features was based on box and whiskers plots, grouping values of patients with the same class of *RAP*. The Fisher ratio (i.e., the square of the difference of the means divided by the sum of variances of pairs of distributions) was used as a measure of linear discrimination of two distributions of features.

The operator measurements of IVC size and pulsatility were compared with the semi-automated estimations using scatter and Bland–Altman plots.

The performance of the continuous estimation of *RAP* of the *LM* was quantified by the mean absolute error in mmHg. Moreover, a scatter plot was used to explore the estimation error. The performances of the classifiers (*SVM* and *LM*, the latter after output splitting into 3 classes) were quantified in terms of *Sensitivity*, *Specificity*, *Precision*, *Accuracy*, and *F-score*, defined as

(1) $$Sensitivity=\frac{TP}{TP+FN}\;Specificity=\frac{TN}{TN+FP}\;Precision=\frac{TP}{TP+FP}$$

(2) $$Accuracy=\frac{TP+TN}{FP+FN+TP+TN}\;F-score=\frac{2\cdot Precision\cdot Sensitivity}{Precision+Sensitivity}$$

where TP, TN, FP and FN indicate the number of true positives, true negatives, false positives, and false negatives, respectively (one class against the other two), reported in confusion matrixes.

## 3. Results

Among the 212 patients randomly selected from the FTGM cohort (range of recruitment: 28 March 2012–27 April 2021), 42 patients were excluded due to technical issues (IVC not visualized by echocardiography, or poor image quality due to the presence of shadow cones). Notice that the drop out is about 20%, which is in line with other studies on IVC. Consider also that the videos were not acquired with the aim of carrying out the edge tracking (as was the case in a precedent study by our group, in which we still discarded the 20% of cases [30]). Finally, 170 patients composed the final dataset, as indicated in Table 2.

Each patient was characterized by the two IVC estimated parameters (CCI and mean diameter) together with other standard clinical and echocardiographic features, as indicated in Table 1.

**Figure 1:**
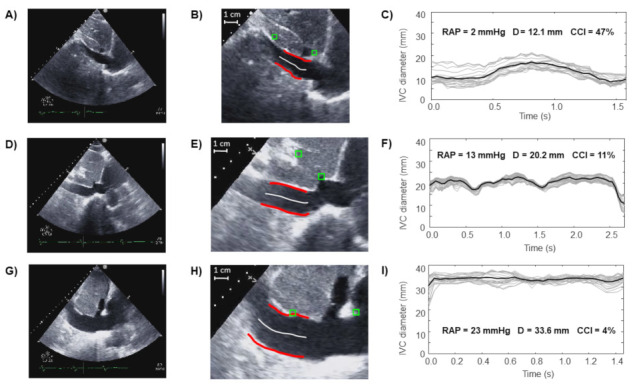
Examples of US video frames of three subjects (**A**–**I**), IVC borders and midline highlighted in red and white, respectively, (**B**,**E**,**H**) and vessel diameters over time (**C**,**F**,**I**) obtained by the semi-automated algorithm, together with the values of CCI, mean diameter, and RAP. The three subjects belong to different RAP classes: low (**A**–**C**), intermediate (**D**–**F**), or high (**G**–**I**).

Figure 1 shows a few examples of data from patients with different values of RAP: higher values of RAP were associated to IVC with greater size and smaller pulsatility.

**Figure 2:**
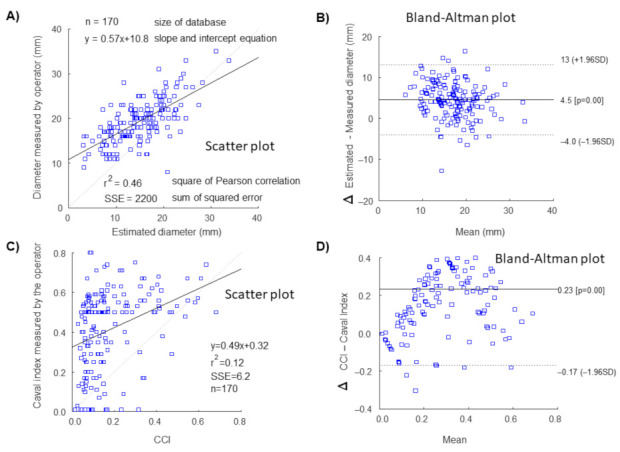
Comparison of IVC properties measured by the operator or estimated by the semi-automated algorithm. (**A**) Scatter plot and (**B**) Bland–Altman representation of IVC size. (**C**) Scatter plot and (**D**) Bland–Altman representation of IVC pulsatility (in terms of caval index after sniff measured by the operator and *CCI* obtained from short US videos).

A comparison between IVC features either measured by the operator or estimated automatically by the algorithm is provided in Figure 2. Scatter and Bland–Altman plots are shown for IVC diameter and pulsatility. A lower correlation and a wider variability between the two methods were observed for IVC pulsatility compared to IVC diameter. Notice that the caval index measured by the operator was induced by sniff maneuver, while CCI automatically calculated by the software only considering oscillations induced by heartbeats.

**Figure 3:**
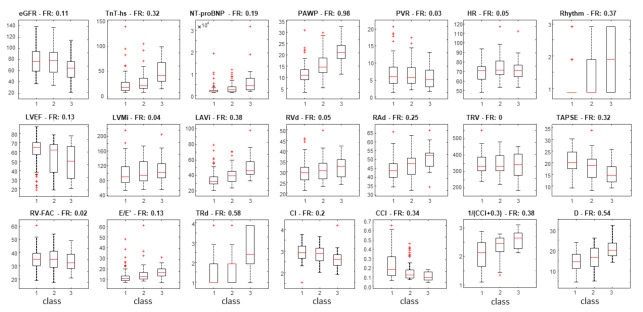
Box and whisker plots of the different features, dividing the dataset in the 3 RAP classes (1 = low, 2 = intermediate, 3 = high). The average of Fisher ratios (FR) is also indicated. CI: cardiac index; CCI: cardiac caval index; eGFR: estimate glomerular filtration rate; hs-TnT: high sensitivity troponin T; D: inferior vena cava diameter; LAVI: left atria volume index; LVEF: left ventricular ejection fraction; LVMI: left ventricular mass index; NT-proBNP: amino terminal pro brain natriuretic peptide; PAWP: precapillary artery wedge pressure; PVR: pulmonary vascular resistances; RAd: right atrial diameter; RVd: right ventricular diameter; RV-FAC: right ventricular fractional area change; TAPSE: tricuspid annular plane excursion; TVR: tricuspid regurgitation velocity; TRd: tricuspid regurgitation degree.

The distributions of the different features of patients belonging to different classes of RAP are shown in Figure 3. Notice that there are large variations among patients with overlapping distributions. However, many features show a clear trend of the median, e.g., an increasing IVC diameter and a decreasing CCI for higher values of RAP.

**Figure 4:**
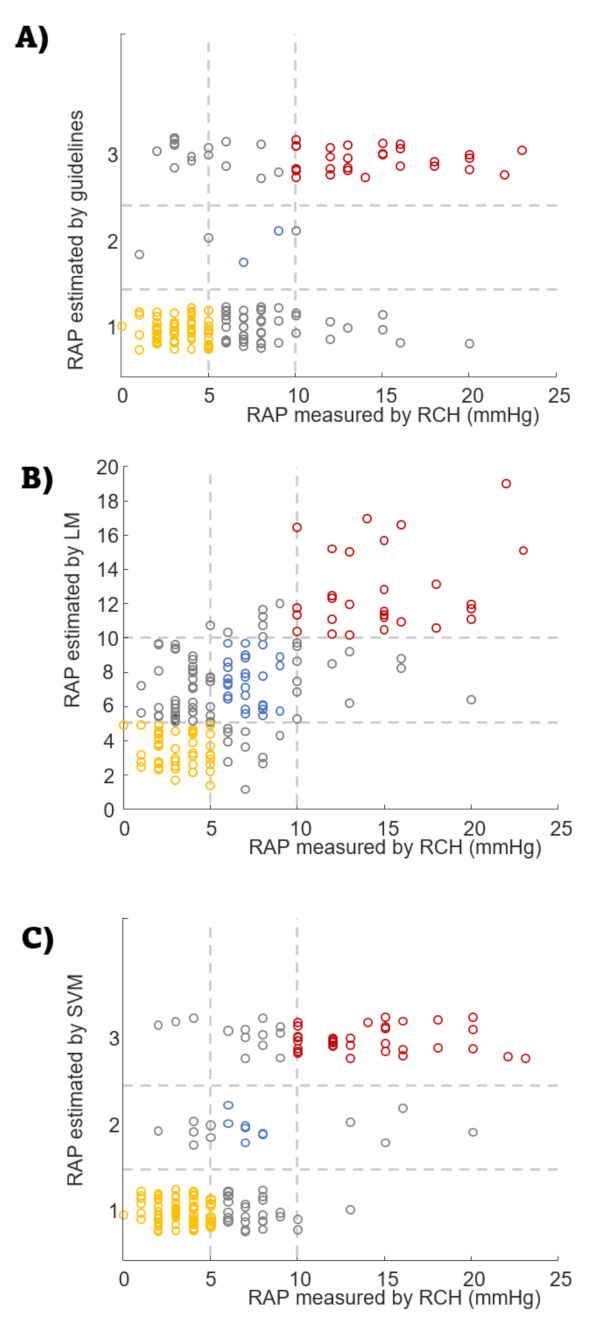
Classification of the subjects obtained by (**A**) guidelines, (**B**) LM, and (**C**) SVM. To avoid superposition of different cases, the estimated values are randomly shifted around the categorical output provided by guidelines and SVM (on the other hand, the continuous output of the linear model is shown in (**B**) as a scatter plot). RAP classes are: 1 = low, 2 = intermediate, 3 = high. LM: linear model; RAP: right atrial pressure; RHC right heart catheterization; SVM: support vector machine.

Figure 4 shows the values of measured *RAP* and the estimated classes obtained by guidelines and by the best selected *LM* and *SVM* classifiers. The selected LM is given by the following equation:

(3) $$RAP=0.26+11.59\frac{PAPW}{33.7}+3.89\frac{TAPSE}{35}-8.28\frac{E/E^{\prime }}{30}-2.3\frac{TRd}{4}+\frac{3.69}{3}\frac{1}{CCI+0.3}+2.82\frac{D}{34}$$

The equation includes six features, which are among those with largest average Fisher ratio. Each term is divided by the maximum value: this way, the multiplicative weight allows to compare the effective influence of each term included in the model. The mean of the absolute error in *RAP* estimation is 2.56 mmHg. Considering this average error as the effective resolution of the estimation model, the range of *RAP* of our patients (around 0–24 mmHg) would be split into about nine values: this is an indication of the resolution of our model in the follow-up of a patient. Accuracy is quite similar when the dataset is split in different subgroups: Bland–Altman analysis is shown in the Appendix C for different subgroups, including pulmonary hypertension, as well as patients with worsening/acute HF and dyspnea of unknown origin. Another possible problem can be found in case of not regular rhythm, as RR variability could potentially decrease the precision of the method, as CCI could be affected: this problem is also investigated in the Appendix C, showing that the accuracy of RAP estimation is not biased when considering patients without sinus rhythm (e.g., with atrial fibrillation).

The SVM model selected eight features, which were added to IVC size and pulsatility: TAPSE, PAPW, LVEF, LVMi, E/E${}^{\prime }$, RV-FAC, TRV, and CI (written in order of importance for the backward selection algorithm). Notice that most features included in the LM were also selected by the SVM. Some features have not a good linear discrimination (such as TRV, which has a vanishing average Fisher ratio): nevertheless, the non-linear model used by SVM can exploit their information.

**Figure 5:**
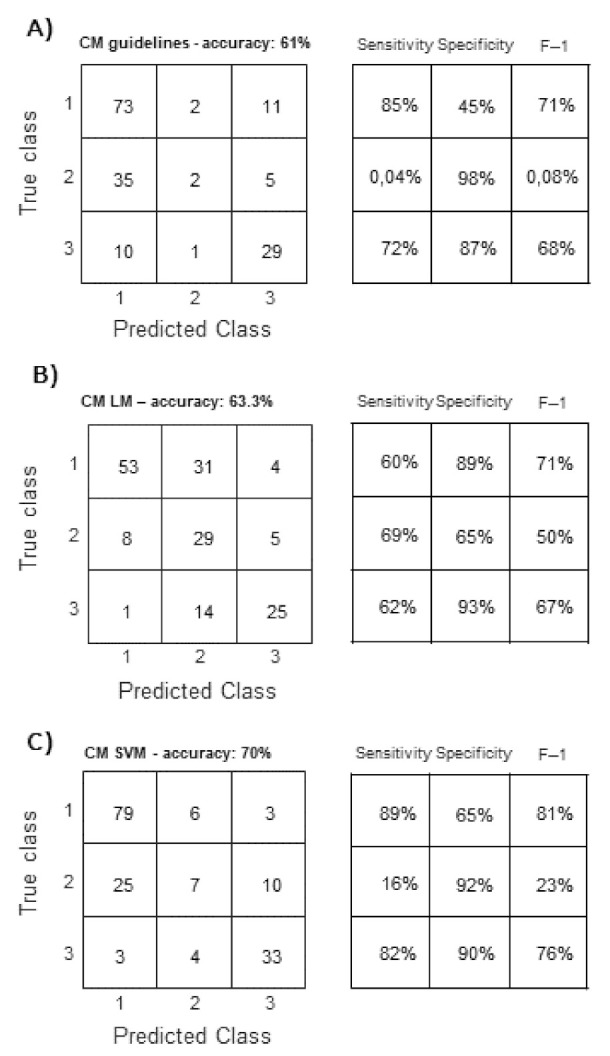
Confusion matrix (CM) of leave-one-out cross validation of (**A**) guidelines, LM (**B**), and SVM model (**C**). RAP classes are: 1 = low, 2 = intermediate, 3 = high. The following average performance indexes were obtained. Guidelines: sensitivity 54.0%, specificity 76.4%, F1 score 49.3%, precision 55.4%. LM: sensitivity 63.7%, specificity 82.2%, F1 score 62.7%, precision 72.6%. SVM: sensitivity 62.3%, specificity 82.3%, F1 score 60.0%, precision 62.2%.

The confusion matrixes of the three considered classifiers, i.e., Guidelines, LM, and SVM, are shown in Figure 5. Different performance indexes are shown or indicated in the caption. Notice that they are all higher when considering the proposed innovative models, than when using the guidelines. Specifically, guidelines algorithm showed a clear limitation in recognizing the intermediate values of RAP.

## 4. Discussion

In the current study, two novel multivariable algorithms (LM and SVM models) based on IVC diameter and CCI automatically calculated by a software (without any respiratory maneuver and with very short recordings, <3 s) and other echocardiographic features were tested for the estimation of RAP in a retrospective cohort of patients undergoing echocardiography and RHC (within 24 h). Both methods showed a higher accuracy as compared to the algorithm proposed by the guidelines [1,7], especially in the intermediate range of RAP (5–10 mmHg), in which the performance of the guidelines seems particularly low.

Echocardiography has long been used as a non-invasive tool to estimate cardiopulmonary hemodynamics [36]. Echocardiography, by exploiting the dynamic combination of 2D images and Doppler-derived metrics, outperforms other imaging modalities in this specific setting. Further advantages of echocardiography are its relatively low-cost, the possibility to be used at bedside even in critically ill inpatients and to be serially repeated during follow-up in outpatients after treatment implementation [37].

In particular, the estimation of RAP by echocardiography would be of great importance in several clinical scenarios, both for diagnostic and prognostic purposes, as well for management of fluid charge/unloading in acute and chronic conditions [26]. Unfortunately, the estimation of RAP is so far burdened by a relatively high inaccuracy and imprecision [38]. The current algorithm, proposed by the Guidelines, only relies on IVC diameter and the caval index obtained by making the subject inspire while recording IVC diameter relative variation either in M-mode or B-mode. This method adds to the physiological variability related to operator dependency of US other sources of variability due to patient capability to perform the inspiratory maneuver [30]. Moreover, RAP is a complex measure that is not only influenced by the central venous compartment, but obviously also by heart function and compliance [25].

The use of automated approaches to extract information on IVC size and pulsatility could overcome some of the above-mentioned limitations. Moreover, the integration of other measurements obtained by echocardiography could help in refining RAP estimation. Our work explored these two directions, providing some promising results. Specifically, the automated processing of IVC video clips have been proposed to remove part of the subjectivity in the assessment of IVC properties [39]. Moreover, tracking the respirophasic movements of IVC [40] allows to investigate the same section in different frames (which is not possible in M-mode and arbitrarily chosen by the operator when working in post-processing on B-mode frames). Our automated processing allows, also, to average across a portion of the IVC [27], making the characterization more reliable and repeatable [34].

However, many problems are still present and may have a great effect on our measurements. For example, the geometry of IVC is very complicated [25]. The cross-section is usually far from being circular [28], and the long axis view considered in this study cannot account for this specific vascular geometry. Out-of-plane movements can also induce a variation of section measured along a single plane [41]. Furthermore, different modalities of breathing may generate different pressure distributions in the thorax and abdomen, inducing different pulsatility of the IVC [42]. For this reason, a collapsibility index related only to the heartbeat as the CCI has been proposed [43]. Recently, it was shown to have good stability and to provide consistent information on fluid responsiveness [35]. However, there are many parameters affecting hemodynamics that are not measured (e.g., compliance of vessels and surrounding tissues, abdominal pressure variations, geometrical details, blood rheology, etc.) and influence our estimations. Specifically, many variables affect IVC size and pulsatility, such as its anatomy (so that a large size could be due either to a large internal pressure or to the subject having an IVC with great cross-section), the stiffness of its borders, or the compliance of the external tissues [25]. Indeed the addition of other echocardiographic features to IVC size and CCI showed a significant improvement of the model only based on IVC size and collapsibility. Their linear discrimination capability was first explored, splitting echocardiographic values among subjects having either a low, medium, or high values of RAP. PAPW, TRd, and IVC size were the three measurements that better discriminated among those three classes of RAP. Thus, it emerges that a good estimation of RAP requires information from both heart chambers and the IVC. An automated method (the backward elimination) was then employed to select the most important features for our models. Those included in the LM had also good linear discrimination ability. Some additional features were included by the SVM that, making a non-linear processing, extracted information also from measurements, which did not show good linear discrimination of RAP classes. Notice that the SVM allows a better classification than LM, but the latter provides a continuous estimation of RAP, which could be useful in many conditions, e.g., a follow-up or the analysis of the response of a patient to a treatment. Our methods achieved promising results: accuracy of about 70% in splitting patients in three classes of RAP; mean absolute error in the estimation of the continuous value of RAP of about 2.6 mmHg.

Many insights and other applications could be considered in the future involving different subjects. For example, an important field to be explored is the performance of IVC edge tracking in the study of athletes’ heart. Indeed an interesting IVC collapsibility pattern was documented in this particular group [44] and our method could possibly provide further insight.

However, we note that our proposed technique was born to provide a support in decision-making in all the clinical settings in which continuous invasive monitoring is unavailable. Of note, in intensive care unit, several others (more complex) techniques are currently available to manage fluid therapy, diuretics or inotropes/vasodilators-vasoconstrictors [45]. Likewise, we should stress that our tool could only provide information at the level of the right heart, while other complementary techniques are suggested to study the left heart, to provide a comprehensive assessment of congestion and fluid overload [46].

### Study Limitations

The retrospective nature of our study did not allow to record optimal data for our automated processing. Specifically, as the US scans were short, other important pulsatility indexes reflecting respiration could not be measured (i.e., the caval index and the respiratory caval index [31]). Moreover, even CCI was likely affected by the specific phase of respiration in which the video was recorded (a stable measurement is obtained by averaging the CCI in a complete respiration cycle [35]). A further relevant problem is due to the lack of feedback in real time by the operator performing the US acquisition. Prospective studies are needed to make our processing algorithms extract the best information and to explore this promising possibility of further improving the accuracy of non-invasive and automatic echocardiographic estimation of RAP.

Finally, in most non-invasive studies, echocardiographic and invasive assessment are performed within 6 h [30]. In our center, due to the intense clinical activity, this time interval was wider, introducing a possible bias. However, as stated before, no diuretic, inotropes, vasodilator, or fluid therapy was administered during this time interval, minimizing the variability of key parameters between echo and RHC derived data in the current study.

## 5. Conclusions

The application of a semi-automated method of RAP estimation, based on IVC size and cardiac pulsatility, as well as other key standard echocardiographic features, was shown to be feasible even in low-quality and short acquisition recordings, as demonstrated in this relatively large retrospective study. Both LM and SVM algorithms derived by IVC edge-tracking outperformed the current guidelines, especially in cases of intermediate RAP values. If confirmed by larger studies with multicentric and prospective design, this work will open to the use of a novel valuable tool with a high accuracy and reproducibility, widening (e.g., to nurses) and making more reliable the RAP estimation in several clinical conditions in the future.

## 6. Patents

An instrument implementing the algorithms for IVC delineation used in this paper was patented by Politecnico di Torino and Universitá di Torino (WO 2018/134726).

## Figures and Tables

**Table 1:** Features included in this study, with indication of the number of missing values (170 patients selected).

Feature	Abbreviation	Number of Missing Values
**Rhythm**		
Heart rate	HR	1
Rhythm (3 classes: sinus rhythm/atrial fibrillation/pacing)	Rhythm	0
**Left chambers**		
Left ventricular ejection fraction	LVEF	0
Cardiac index	CI	6
Left ventricular mass index	LVMi	2
E/E${}^{\prime }$	E/E${}^{\prime }$	0
Pulmonary artery wedge pressure	PAWP	0
Left atrial volume index	LAVi	1
**Pulmonary circulation**		
Pulmonary vascular resistance	PVR	0
Tricuspid regurgitation velocity	TRV	0
**Right chambers**		
Right atrial diameter	RAd	0
Right ventricular diameter	RVd	1
Tricuspid annular plane excursion	TAPSE	3
RV fractional area change	RV-FAC	0
Tricuspid regurgitation degree	TRd	0
Cardiac caval index	CCI	0
Mean IVC diametr	IVCd	0
**Biomarkers**		
Amino-terminal pro-B-type natriuretic peptide	NT-proBNP	2
Troponin-T (high sensitivity) TnT-hs	TnT-hs	4
Estimated Glomerular Filtration Rate	eGFR	2

**Table 2:** Characteristics of the population included in our dataset. CI: cardiac index; CCI: cardiac caval index; eGFR: estimate glomerular filtration rate (mdrd equation); hs-TnT: high sensitivity troponin T; IVCd: inferior vena cava diameter; LAVI: left atria volume index; LVEF: left ventricular ejection fraction; LVMI: left ventricular mass index; NYHA: New York Heart Association; NT-proBNP: amino terminal pro brain natriuretic peptide; PAWP: precapillary artery wedge pressure; PVR: pulmonary vascular resistances; RAd: right atrial diameter; RVd: right ventricular diameter; RV-FAC: right ventricular fractional area change; TAPSE: tricuspid annular plane excursion; TVR: tricuspid regurgitation velocity; TRd: tricuspid regurgitation degree.

Age (years)	64 ± 14
**Gender**	
Female (%)	55.3
Male (%)	44.7
**Clinical referral for RHC**	
Pulmonary arterial hypertension (%)	42
Worsening/acute heart failure (%)	25
Evaluation of dyspnea of new onset (%)	22
Valvular heart disease (%)	4
Other (%)	7
**Heart failure with reduced EF (%)**	29
**Risk factors and comorbidities**	
Systemic arterial hypertension	40
Diabetes	22
Dyslipidemia	22
Chronic obstructive pulmonary disease	17
Chronic kidney disease	15
**NYHA class**	
I (%)	20
II (%)	45
III (%)	31
IV (%)	4
**Rhythm**	
Heart rate	72 ± 13
Sinus rhythm (%)	65
Atrial fibrillation (%)	28
Pacing (%)	7
**Left chambers**	
LVEF (%)	52 ± 18
CI (L/min/mq)	2.7 ± 0.7
LVMI (g/mq)	103 ± 36
E/E${}^{\prime }$	12 ± 8
PAWP (mmHg)	15 ± 7
LAVi (mL/mq)	39 ± 14
**Pulmonary circulation**	
PVR (w.u.)	3.3 ± 2.2
TRV (cm/s)	336 ± 65
TRd	1.876 ± 0.864
**Right chambers**	
RAd (mm)	46 ± 8
RVd (mm)	29 ± 5
TAPSE (mm)	19 ± 6
RV-FAC (%)	35 ± 9
CCI (%)	42 ± 20
IVCd (mm)	19 ± 5
**Biomarkers**	
NT-proBNP (ng/L)	785 (231–2429)
hs-TnT (ng/L)	16.34 (8.57–30.52)
eGFR (mL/min/1.73 mq) MDRD	74.4 ± 26.9
Serum creatinine (mg/dL)	0.98 ± 0.38

**Figure A1:**
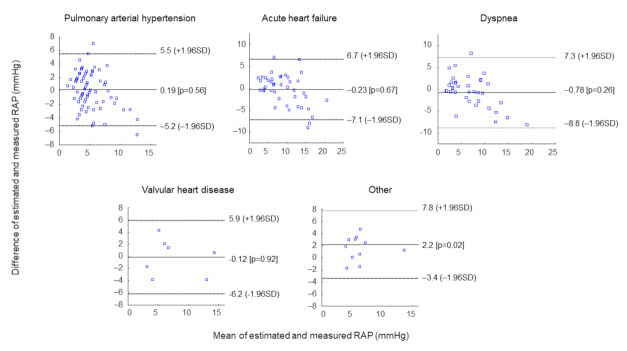
Bland–Altman plots comparing estimated (by LM) and measured RAP on our dataset split into different groups of patients.

**Figure A2:**
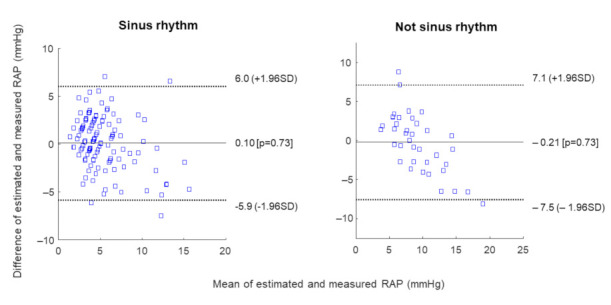
Bland–Altman plots comparing estimated (by LM) and measured RAP on our dataset split into patients with sinus rhythm or with irregular pacing.

## Data Availability

Data are available from the corresponding author upon reasonable request.

## Abbreviations

The following abbreviations are used in this manuscript:

CI	Cardiac index
CCI	Cardiac caval index
eGFR	Estimate glomerular filtration rate
hs-TnT	High sensitivity troponin T
IVC	Inferior vena cava
IVCd	Inferior vena cava diameter
LAVI	Left atria volume index
LVEF	Left ventricular ejection fraction
LVMI	Left ventricular mass index
NYHA	New York Heart Association
NT-proBNP	Amino terminal pro brain natriuretic peptide
PAWP	Precapillary artery wedge pressure
PVR	Pulmonary vascular resistances
RAd	Right atrial diameter
RAP	Right atrial pressure
RVd	Right ventricular diameter
RV-FAC	Right ventricular fractional area change
TAPSE	Tricuspid annular plane excursion
TRd	Tricuspid regurgitation degree
TVR	Tricuspid regurgitation velocity
US	Ultrasound

## Appendix A. Echocardiographic Assessment

Non-invasive estimations of the following hemodynamic parameters were obtained with standard clinical approaches: RAP, systolic pulmonary artery pressure (sPAP), stroke volume (SV), and cardiac output (CO). RAP was estimated according to latest guidelines, both from inferior vena cava (IVC) diameters and from the collapsibility index. Maximum and minimum diameters during the respiratory cycle were considered, usually corresponding to inspiration and expiration, respectively; IVC diameter variations during the cardiac cycle were not considered. A diameter greater than 2.1 cm and a collapsibility index lower than 50% were used to distinguish three ranges of values [normal (0–5 mmHg), intermediate (5–10 mmHg), and elevated (10–20 mmHg) RAP]; LV E/E${}^{\prime }$ was calculated employing annular velocities at the septal wall and E wave velocity of the pulse wave Doppler at the level of the mitral leaflets and annular velocities measured by PW TDI at the level of the interventricular septum (four chamber view) at the basal level. PAPs was estimated using the maximum velocity of tricuspid regurgitation (*TRV*) and the echocardiographic estimation of *RAP*, applying the modified Bernoulli’s equation

(A1) $$sPAP=4TRV^{2}+RAP$$

where *RAP* is the mean value of the class estimated by the guidelines. *SV* was estimated using the diameter of the outflow tract of the left ventricle (dLVOT) to calculate the cross-sectional area (*CSA*), and the time velocity integral (*TVI*) *LVOT*, with the following formula

(A2) SV=CSA·TVILVOT

CO was finally calculated by multiplying SV by HR.

## Appendix B. Right Heart Catheterization

The invasive hemodynamic study was performed by a brachial, femoral. or jugular venous approach, based on the choice of the operator. The Swan Ganz catheter was advanced with an inflated balloon until the capillary wedge position was reached. Then the average capillary pressure was recorded. By subsequent deflation of the balloon, the pulmonary pressures (systolic, diastolic, and mean) and then the RAP (by the appropriate channel) were recorded. The classical morphology of the pressure wave had to be recognizable (two positive deflections, a and v, and two negative deflections, x and y) when evaluating the RAP. Subsequently the cardiac output was measured. If pharmacological interventions were performed during the hemodynamic study, the RAP was measured before. The transduction system was zeroed at the mid-thoracic level, before the measurement of intracavitary pressure.

## Appendix C. Further Results

Figure A1 shows Bland–Altman analysis of our dataset after splitting patients in specific subgroups. Accuracy is quite similar in the different subgroups (with a bias found in the group of Other Pathologies, but the number of patients included is fairly small).

Figure A2 shows Bland–Altman analysis of our dataset separating patients with sinus rhythm from those showing an irregular rhythm. Accuracy is quite similar in the two subgroups.
